# An anatomic checklist for accurate staging of grossly invasive thyroid cancer

**DOI:** 10.1530/ETJ-24-0289

**Published:** 2025-03-03

**Authors:** Mark L Urken, Margaret Brandwein-Weber, Raymond L Chai, Mark Zafereo, Maisie Shindo, Joseph Scharpf, Jun Fan, Alex Silberzweig, Justin K Joseph, Ronald Ghossein, Ashok Shaha, Zubair Baloch, R Michael Tuttle

**Affiliations:** ^1^ Department of Otolaryngology Head and Neck Surgery, Icahn School of Medicine at Mount Sinai, New York, USA; ^2^ Thyroid, Head and Neck Cancer (THANC) Foundation, New York, USA; ^3^ Department of Pathology, Icahn School of Medicine at Mount Sinai, New York, USA; ^4^ Department of Head & Neck Surgery, MD Anderson Cancer Center, Houston, USA; ^5^ Department of Otolaryngology – Head & Neck Surgery, Oregon Health & Science University, Portland, USA; ^6^ Head and Neck Institute, Cleveland Clinic Main Campus, Cleveland, USA; ^7^ Department of Pathology and Laboratory Medicine Memorial Sloan-Kettering Cancer Center, New York, USA; ^8^ Department of Surgery, Memorial Sloan-Kettering Cancer Center, New York, USA; ^9^ Department of Pathology and Laboratory Medicine, University of Pennsylvania Medical Center, Philadelphia, USA; ^10^ Department of Medicine, Memorial Sloan-Kettering Cancer Center, New York, USA

**Keywords:** invasive thyroid cancer anatomic checklist

## Abstract

**Objective:**

The final surgical pathology report follows the patient throughout their cancer journey. For locoregionally advanced cancers, lack of surgeon-pathologist communication can lead to understaging, adversely impacting management. Our study aims to improve the accuracy of staging grossly invasive thyroid cancer by introducing an anatomic checklist, enhancing surgeon-pathologist communication.

**Methods:**

We studied 35 consecutive patients with either gross extrathyroidal or extranodal extension, 29 of whom underwent primary resections requiring AJCC staging. Surgeon A initially only dictated an operative report. Surgeon B transmitted an anatomic checklist to the pathologist in addition to the standard operative note. Final pathology reports were reviewed for AJCC staging accuracy. Surgeon A transitioned to submission of an anatomic checklist for his final six cases.

**Results:**

13 of the 14 final pathology reports without a checklist were understaged. All 15 cases with a surgeon completed anatomic checklist were accurately staged. There was a statistically significant improvement in the accuracy of staging reported in the final pathology reports when an anatomic checklist was submitted as compared to when it was not (*P* < 0.01, Fisher exact test, two-tailed). All final pathology reports for recurrent cases without a checklist failed to define the anatomic parts that were resected. The time to complete the checklist was less than 90 s.

**Conclusion:**

A surgeon-completed anatomic checklist allows pathologists to more accurately stage grossly invasive thyroid cancers. This rapidly completed form eliminates the need for pathologists to analyze the operative note and facilitates both risk of recurrence and AJCC stage determination.

## Introduction

The American Joint Commission on Cancer (AJCC) cancer staging system stratifies patients according to disease-specific mortality risk. This static determination is based on initial tumor characteristics (size/extent of the primary tumor, regional lymph node status and distant metastasis) plus clinical information included up to 4 months after surgery. The current AJCC schema is not applicable in the setting of recurrent surgical disease. Risk stratification for disease recurrence is clinically more relevant for thyroid cancer patients than predicting disease-specific mortality, which is generally low. The American Thyroid Association (ATA) risk of recurrence (ROR) provides a framework for predicting a patient’s risk of recurrent disease (1, 2).

AJCC thyroid cancer staging acknowledges that gross extrathyroidal extension (ETE) identified during surgery is a key prognosticator and offers the following guidance: ‘The surgeon’s description of gross ETE must also be included’ (3). AJCC staging incorporates all pertinent information from preoperative evaluations, intraoperative findings, postoperative pathology, as well as pertinent information that becomes available up to 4 months after surgery (3). The Surgical Affairs Committee of the ATA highlights the importance of communicating intraoperative surgical findings. The operative report is the only current standard for conveying this information. However, it is time-consuming and challenging for pathologists, endocrinologists, nuclear medicine specialists and radiation oncologists to glean important anatomic details from these reports (4, 5). When one depends on scouring the surgeon’s operative report, there are numerous potential pitfalls that can lead to understaging and inaccurate AJCC and ATA-ROR determinations.

We address this information gap by presenting a newly developed checklist, which includes anatomic figures that advise pathologists on the anatomic details of intraoperative findings. This level of detail not only improves staging but also helps in planning for adjuvant radiation therapy and interpreting postoperative imaging. It can also improve our understanding of clinical scenarios currently lumped together as pT4a disease. For instance, involvement of the RLN, esophagus and laryngotracheal complex is all similarly staged as pT4a. It is unclear as to whether these different types of structural involvement could be associated with different outcomes. The additional anatomic detail in the checklist is intended to supplement, but not replace, the information included in the College of American Pathologists thyroid protocol and will facilitate outcomes research (6).

The absence of an AJCC staging system for recurrent disease makes formal reporting in this setting challenging and nonuniform. The rationale for including a checklist for recurrent disease is that anatomic organs are more frequently resected. Gross extranodal extension (ENE) involving anatomic structures should be accurately identified in the pathology report. Distinguishing recurrent disease that involves lymph nodes from structural recurrence involving nerves, bone, vessels, the laryngotracheal complex and the esophagus is important, but there is currently no mechanism for doing so.

In recognition of the importance of ease of checklist completion in order to support surgeon and pathologist adoption, both an online and a printed version of the form (https://tiro.expert/analysis-tools) are readily available. The purpose of this study is to validate the effectiveness of the anatomic checklist in supporting both AJCC and ATA-ROR staging.

## Materials and methods

**Figure 1:**
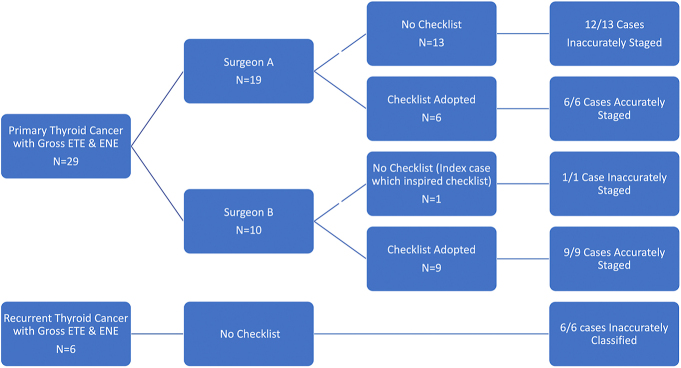
A breakdown of reported cases with associated information regarding the inclusion or omission of the checklist and the results of accuracy of staging/classification.

This retrospective study has obtained IRB approval from the Icahn School of Medicine at Mount Sinai, New York (STUDY-18-01236). Over a 24-month period, two senior head and neck surgeons (A and B) tracked the accuracy of final staging in a consecutive cohort of both primary and recurrent thyroid cancer patients that demonstrated clinically gross invasion of surrounding structures. Only cases with ETE or ENE were included in this study. Surgeon A used the checklist for the last six of 19 primary cases but not for the first 13. Surgeon B did not use the checklist for the first case, which served as the impetus for this study. The checklist was developed and utilized for surgeon B’s next nine cases (Fig. 1). Before sign-out, the pathologist searched the surgeon’s operative note in cases without a checklist submission. We compared the accuracy of final pathology staging on primary resections submitted for both surgeons. The checklist includes anatomic details regarding the laryngotracheal complex, nerves, muscles, bone, vasculature and pharyngoesophagus, which represent the sites usually involved by gross ETE or ENE.

Preoperative clinicopathologic information related to the results of molecular testing and the function of the vocal cords is often known by the surgeon and may be included in a clinic record but is not traditionally included in an operative note. These vital pieces of information are incorporated in the anatomic checklist as line items to be completed.

### The anatomic checklist

**Figure 2:**
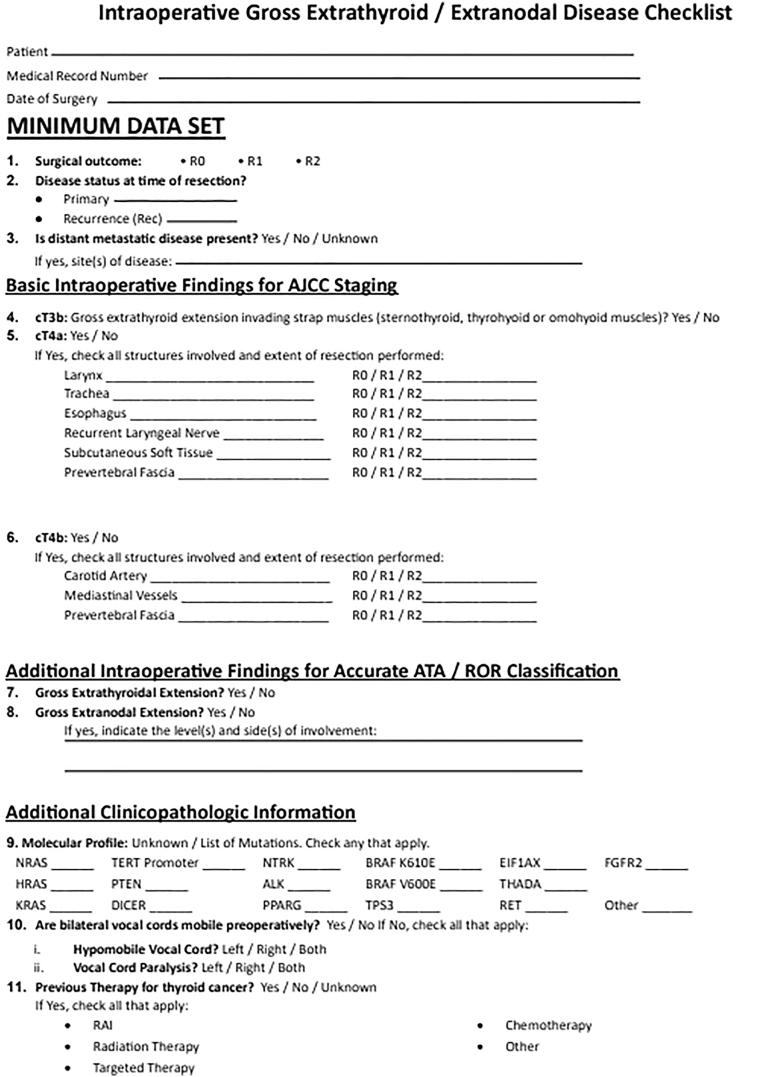
Intraoperative gross extrathyroidal/extranodal disease checklist. The checklist is divided into sections to capture key data points necessary for accurate AJCC staging and ATA ROR classification. It includes fields for documenting preoperative clinicopathological findings, surgical outcomes, disease status and presence of distant metastatic disease.

**Figure 3:**
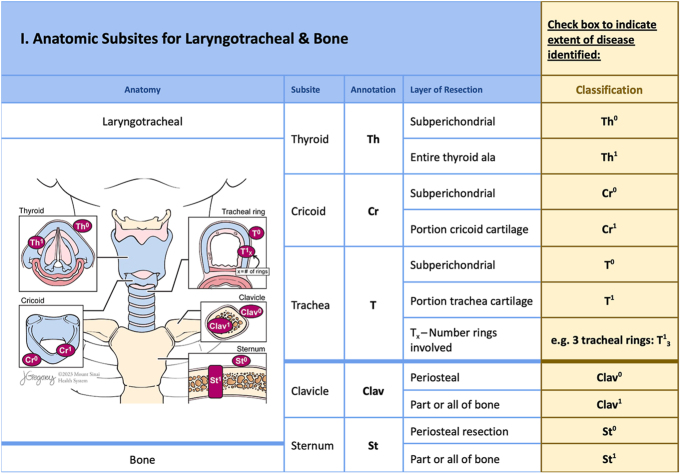
The second part of the anatomical checklist shows the laryngotracheal complex (LT-), indicating the thyroid (Th), cricoid (Cr) and trachea (T) subsites with delineation of the layers of resection, either subperichondrial or full thickness. The surgeon can specify the number of tracheal rings involved (T_x_). In addition, it includes bony structures (B-), such as the clavicular heads (Clav) and sternum (St), indicating whether the involvement is superficial (within the periosteal plane) or necessitates partial or complete resection of the bone.

**Figure 4:**
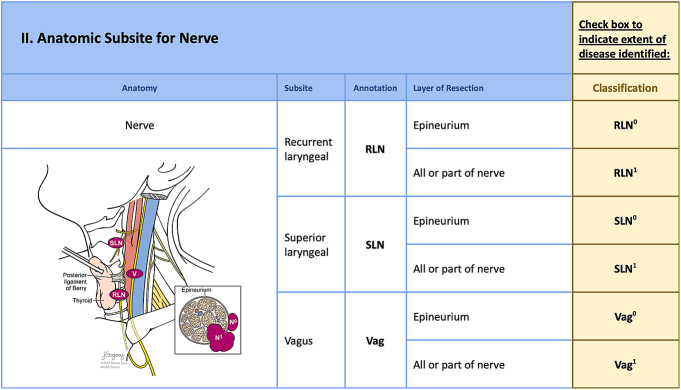
The third part of the checklist illustrates cervical nerve involvement (N-), highlighting the recurrent laryngeal (RLN), superior laryngeal (SLN) and vagus (Vag) nerves. Each nerve subsite is further classified to indicate the layer of resection. The classification system includes RLN^0^, SLN^0^ and Vag^0^ for cases where the nerve can be preserved through subepineural dissection, and RLN^1^, SLN^1^ and Vag^1^ for more substantial invasion requiring nerve sacrifice.

**Figure 5:**
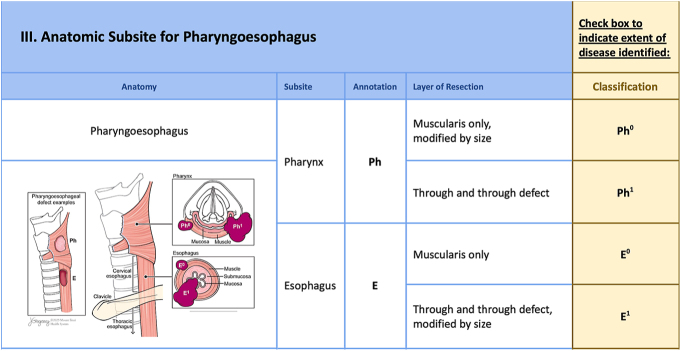
The next part of the checklist allows reporting of involvement of the pharyngoesophagus (PE-), including the pharynx (Ph) and esophagus (E) subsites, detailing classifications for depth of invasion: muscularis only (Ph^0^ and E^0^) and full thickness including mucosa (Ph^1^ and E^1^).

**Figure 6:**
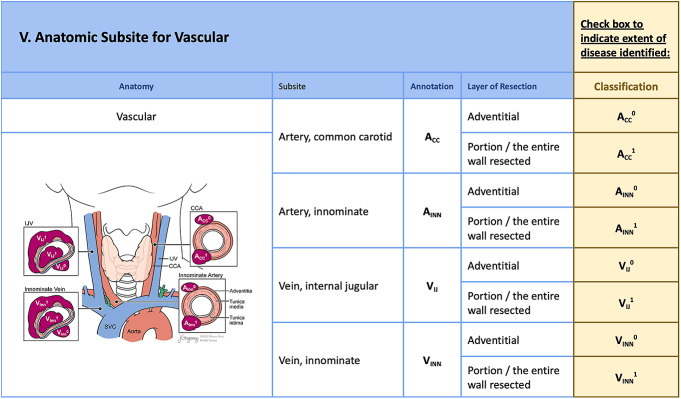
Involvement of vascular structures (VAS-), including the major arteries and veins, is highlighted in the next part of the checklist: the common carotid artery (A_CC_), innominate artery (A_INN_), internal jugular vein (V_IJ_) and innominate vein (V_INN_). The classification system delineates the degree of vessel involvement – from dissection in the subadventitial plane (superscript^0^) to complete transection or encasement (superscript^1^).

**Figure 7:**
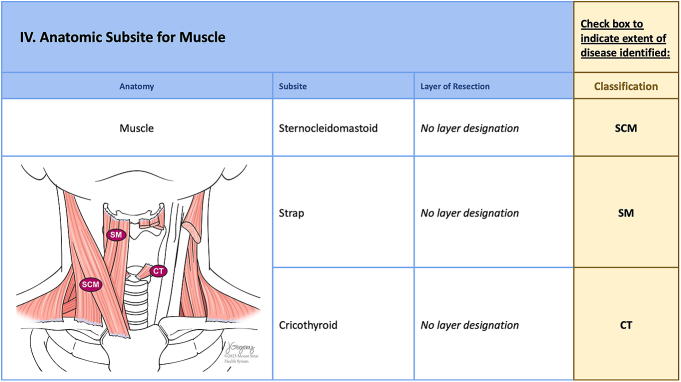
The next part of the anatomic checklist shows involvement of the muscles of the neck (M-), including the sternocleidomastoid (SCM), strap (SM) and cricothyroid (CT) muscles. Muscle classification is binary, with no subclassification as to the degree of muscle involvement.

This anatomic checklist (Figs 2, 3, 4, 5, 6, 7) includes the currently accepted details integrated into the AJCC staging system (8th edition) for thyroid cancer (3) and the information needed for ATA-ROR classification (2), reported in the ‘minimum data set’ section of the checklist (Fig. 2). The information included in the checklist evolved out of critical analysis, feedback, modification and consensus-building among highly experienced senior thyroid surgeons and pathologists of the authorship. The checklist facilitates immediate, efficient and accurate AJCC and ATA-ROR staging rather than requiring the pathologist to obtain critical information by reading the operative note or calling the surgeon directly.

Here, we describe the definitions and formatting of this classification system. The base initials used in the classification system designate the anatomic structure (e.g. T signifies trachea), and the superscript designates the degree of invasion. The superscript of either (^0^) or (^1^) indicates the extent of surgery performed on that structure. Generally, the designation (^0^) indicates that the structure was involved by the cancer but dissected in a plane not requiring sacrifice (i.e. epineural layer, perichondrial layer and adventitial layer). There are two exceptions to this convention. For the pharyngoesophagus, if the muscularis is resected and the lumen is not entered, then the designation is also superscript (^0^) (e.g. Ph^0^ and E^0^). Alternatively, the superscript (^1^) reflects that a portion or all of that structure was resected for tumor clearance and resulted in a transmural visceral defect. The involvement of various cervical muscles is a binary determination and does not require a superscript as to the degree of involvement.

### Laryngotracheal complex (LT-)

Laryngotracheal defects (LT-) are further subclassified into thyroid alae (Th), cricoid cartilage (Cr) and trachea (T), which are resected either in a subperichondrial plane (Th^0^, Cr^0^ and T^0^) or in full thickness (Th^1^, Cr^1^ and T^1^). The subscript T_x_ indicates the number of tracheal rings involved (Fig. 3).

### Bone (B-)

Although rare, the clavicular heads (Clav) and sternum (St) are bony structures that can be involved. For example, B-Clav^0^ indicates disease removed from the clavicular head in a subperiosteal plane. Resection of bone from the sternum is designated B-St^1^. (Fig. 3).

### Nerves (N-)

The recurrent laryngeal (RLN), superior laryngeal (SLN) and vagus (Vag**)** nerves are the most frequently involved. Nerve involvement is either epineural (N-RLN^0^), amenable to nerve preservation, or more substantial invasion (N-RLN^1^) requiring sacrifice (Fig. 4).

### Pharyngeal/esophageal defects (PE-)

**Figure 8:**
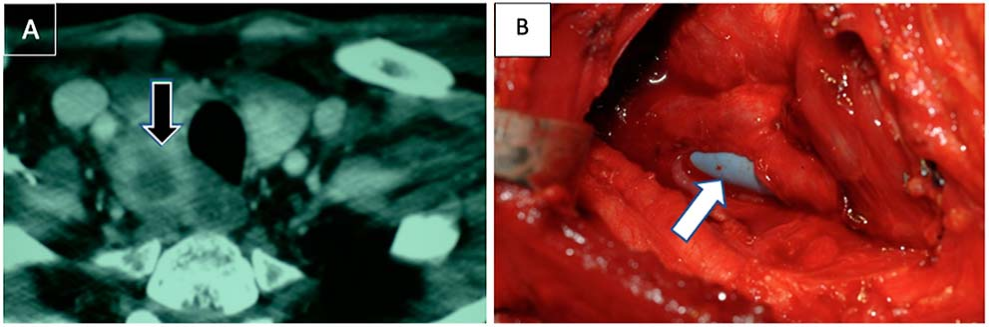
(A) Recurrent thyroid cancer in a central compartment lymph node (black arrow). (B) Resection required removal of the RLN as well as a portion of the cervical esophageal wall, leading to the creation of a transmural defect amenable to primary repair performed over a bougie seen in the lumen of the esophagus (white arrow). Defect classified as **Rec N-RLN**^**1**^ **PE-E**^**1**^.

**Figure 9:**
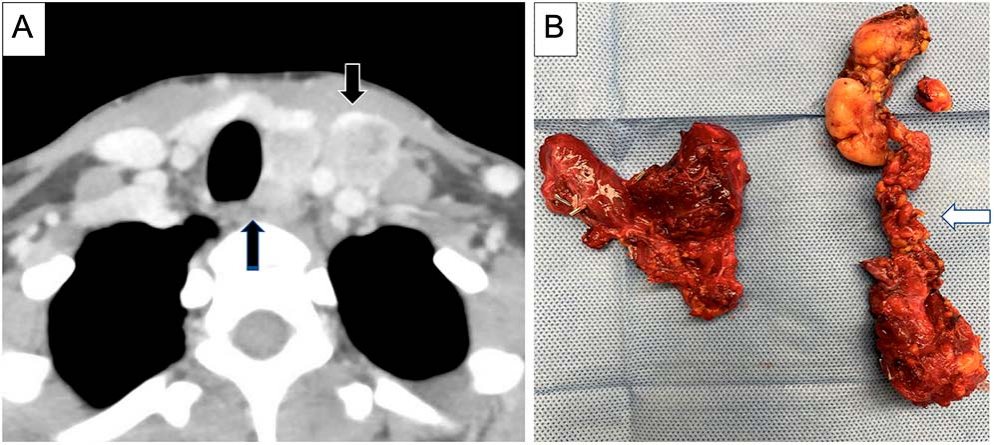
(A) Primary resection of invasive thyroid cancer with involvement of the internal jugular vein (black arrow, white trim) and the cervical esophageal musculature (solid black arrow), as well as the left RLN. (B) The specimen included a total thyroidectomy and central compartment lymph nodes that were removed in a subperichondrial plane involving two tracheal rings. Lateral compartment resection required resection of the internal jugular vein (white arrow). AJCC staging combined with anatomic information: pT4a N1b Mx PE-E^0^ Vas-V_IJ_^1^ LT-T_2_^0^ N-RLN^1^.

Pharyngoesophageal (PE-) invasion is classified by site and degree of invasion (muscularis only versus muscularis extending to mucosa) (Fig. 5). Pharyngeal invasion limited to the muscularis is classified as Ph^0^. Full-thickness invasion extending into the mucosa is classified as Ph^1^. Esophageal defects are similarly classified: muscularis only (PE-E^0^) versus extension into the mucosa (PE-E^1^). Examples of anatomic staging are provided (Figs 8 and 9).

### Arteries and veins (Vas-)

Vascular invasion (Vas-), divided between arterial (A) or venous (V) invasion, is designated by vessel name (as subscript) and degree of involvement (Fig. 6). The common carotid (Vas-A_CC_) and innominate (Vas-A_INN_) arteries are independently classified as subscripts due to the management challenges and potential prognostic significance of mediastinal versus cervical vessel involvement. The extent of vessel wall involvement is designated by superscript according to surgical clearance. An artery can be preserved by dissection in the subadventitial plane and is designated (Vas-A_CC_^0^) or by complete arterial transection and is designated (Vas-A_CC_^1^). A_CC_^1^ and A_INN_^1^ include arterial encasement correlating with T4b disease in AJCC (1). Venous involvement is either internal jugular vein (Vas-V_IJ_) or innominate vein (Vas-V_INN_). The extent of vein involvement is classified according to the extent of resection required for surgical clearance (V_IJ_^0^ versus V_IJ_^1^). Venous tumor thrombus, seen in some cases of invasive thyroid cancer, is included in the Vas-V_IJ_^1^ classification.

### Muscle (M-)

Muscle involvement (M-) designates removal of part or all of the strap (SM), sternocleidomastoid (SCM) and cricothyroid (CT) muscles, which are most frequently involved by advanced thyroid cancer (Fig. 7). Gross strap muscle invasion is currently used to classify T3b disease. As noted above, muscle invasion is a binary determination and does not require a superscript.

### Distinguishing surgery for persistent or recurrent (rec) cancer

‘Rec’ followed by the anatomic details of resected structures designates recurrent or persistent disease (Fig. 8 and Table 2).

### Data entry

**Figure 10:**
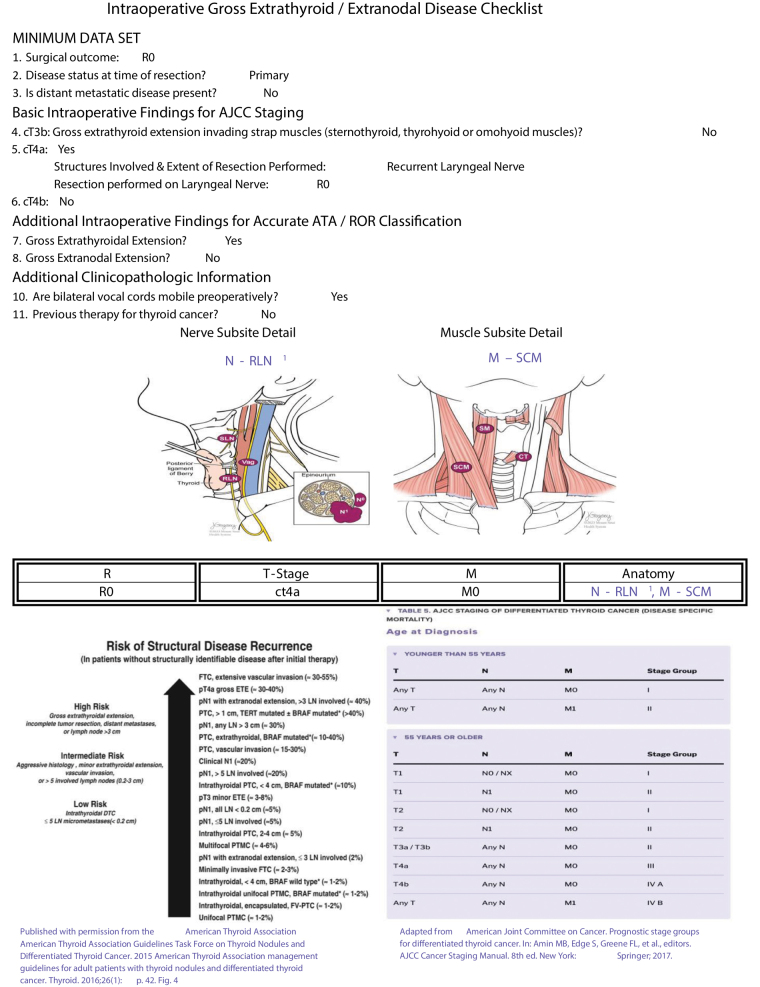
Electronically generated completed checklist output by surgeon B for patient 25. The patient is staged as pT4a N1b Mx N-RLN^1^ M-SCM (sacrifice RLN, resection of the sternocleidomastoid muscle). The checklist includes a minimum data set, basic intraoperative findings per AJCC staging (8th edition) and ATA ROR classifications, clinicopathologic information and a visual representation of nerve and muscle invasion. A table of AJCC staging and ATA ROR is included at the end of each report to help facilitate staging and reporting.

In an effort to enhance the speed and efficiency of postoperative data entry by surgeons, the authors developed two options for accessing and completing the checklist before submitting it to the pathologist. Surgeons can utilize a printed version or an online version of the checklist. Both options are available at https://tiro.expert/analysis-tools. The website allows the surgeon to complete a de-identified electronic version of the checklist, wherein by checking the relevant items, the website automatically produces a final summary of the anatomic designations that can be forwarded to the pathologist and/or entered into the patient’s electronic health record (Fig. 10). In addition to the final anatomic classification, the output form includes both the AJCC and ATA-ROR classifications at the end to make these designations as easy as possible to incorporate into the final pathology report.

The surgeons informally kept track of the time required to complete the anatomic form and submit it to the pathologist.

## Results

During the 24-month interval, 29 primary resections requiring AJCC staging and six recurrent cases were identified. For surgeon A, 12/13 final reports were understaged when an anatomic checklist was not submitted. In one case (#5), the sacrifice of the RLN was clearly stated in the operative report and appropriately noted in the final pathology report. In the latter part of the study period, surgeon A adopted the use of an anatomic checklist, where the final six cases were accurately staged (Fig. 1). For surgeon B, the index resection was understaged (Table 1, patient 20). This led to the development and implementation of this checklist initiative. The remaining nine patients were correctly staged.

**Table 1:** Comparative staging for consecutive primary thyroid cancers with gross invasion by two surgeons.

Surgeon/patient	Checklist submitted	Diagnosis	Reported stage	Items not reported on	Results	Recommended stage and anatomic classification
Surgeon A						
1	No	PDTC	pT3b N0 Mx	PNI, pharynx	UFR	pT4a N0 Mx N-RLN^1^ PE-Ph^1^ (sacrifice RLN, pharyngeal constrictor muscle)
2	No	PDTC	pT1b N1a Mx	Esophagus	UFR	pT4a N1a Mx PE-E^0^ (resection esophageal muscularis)
3	No	PDTC	pT2 N1a Mx	Muscle	UFR	pT3b N1a Mx M-SM (resection strap muscle)
4	No	PTC-FV	pT2 N1a Mx	PNI, pharynx, esophagus, trachea	UFR	pT4a N1a Mx N-RLN^1^ PE-E^0^ LT-T_3_^0^ (sacrifice RLN, resection esophageal muscularis, tracheal perichondrium, three rings)
5	No	PTC-FV	pT1a N1b Mx	RLN resection identified in operative report	NCFR	pT4a N1b Mx N-RLN^1^ (sacrifice RLN)
6	No	PTC-TCV	pT2 N0 Mx	Muscle, esophagus	UFR	pT4a N0Mx M-SM/CT PE-E^0^ (resection strap muscle, cricothyroid muscle, esophageal muscularis)
7	No	PTC-FV	pT3a N0 Mx	Muscle	UFR	pT3b N0 Mx M-SM (resection strap muscle)
8	No	PTC-TCV	pT3a N1a Mx	PNI, muscle, esophagus	UFR	pT4a N1a Mx N-RLN^1^ M-SM PE-E^0^ (sacrifice RLN, resection strap muscle, esophageal muscularis)
9	No	PTC	pT3a N1 Mx	Muscle	UFR	pT3b N1 Mx M-SM (resection strap muscle)
10	No	PTC-TCV	pT3a N0a Mx	Muscle, pharynx, esophagus	UFR	pT4a N0a Mx M-SM/CT PE-E^0^ Ph^0^ (resection strap muscle, cricothyroid, pharyngeal constrictor, esophageal muscularis)
11	No	PTC-TCV	pT2 N0a Mx	Pharynx, esophagus, muscle	UFR	pT4a N0a Mx M-CT PE-E^0^ Ph^0^ (resection cricothyroid muscle, esophageal muscularis, pharyngeal constrictor)
12	No	PTC-TCV	pT2 N1b Mx	Muscle	UFR	pT3b N1b Mx M-SM/CT (resection strap muscle, cricothyroid muscle)
13	No	PTC-TCV	pT4a N1b Mx	Residual cervical gross disease at internal jugular vein & innominate vein	UFR	R2 pT2 N1b Mx VAS-V_IJ/INN_^1^ (unresected gross disease at the junction internal jugular &innominate veins)
14	Yes	PTC-TCV	pT4a N1a M1		NC-AJCC	pT4a N1a M1 N-RLN^1^ LT-T_2_^0^Cr^0^ PE-E^0^Ph^0^ (sacrifice RLN, perichondrium of two tracheal rings, esophageal muscularis, pharyngeal constrictor)
15	Yes	PTC	pT3b N1a Mx		NC-AJCC	pT3b N1a M-SM (resection strap muscles)
16	Yes	PTC	pT4a N0 Mx		NC-AJCC	pT4a N0 Mx N-RLN^0^ (resection RLN epineurium)
17	Yes	PTC	pT4a N0 Mx		NC-AJCC	pT4a N0 Mx N-RLN^1^ PE-Ph^0^E^0^ (sacrifice RLN, resection pharyngeal constrictor, esophageal muscularis)
18	Yes	Anaplastic	pT4a N1b M0		NC-AJCC	R1 pT4a N1b Mx N-RLN^1^ PE-Ph^0^ LT-T_1_^0^ M-SM (sacrifice RLN, resection strap muscle, microscopic residual disease at pharyngeal constrictor, tracheal perichondrium at one tracheal ring)
19	Yes	PTC	PT3b N1a Mx		NC-AJCC	pT3b N1a Mx M-SM (resection strap muscle)
Surgeon B						
20	No	PTC-FV	pT1b N1a Mx	PNI	UFR	pT4a N1a Mx N-RLN^1^ (sacrifice RLN)
21	Yes	FC	pT4a N0 Mx		NC-AJCC	pT4a N0 Mx B-St^1^ Clav^1^ (resection periosteum of sternum, clavicle)
22	Yes	PDTC	pT4a N1b Mx		NC-AJCC	pT4a N1b Mx N-RLN^1^ (sacrifice RLN)
23	Yes	PTC-TCV	pT3b N1b Mx		NC-AJCC	pT3b N1b Mx M-SM (resection strap muscle)
24	Yes	PTC-TCF	pT4a N1b Mx		NC-AJCC	pT4a N1b Mx N-RLN^1^ (sacrifice RLN)
25	Yes	PTC-TCV	pT4a N1b Mx		NC-AJCC	pT4a N1b Mx N-RLN^1^ M-SCM (sacrifice RLN, resection sternocleidomastoid muscle)
26	Yes	PTC-TCV	pT4a N1b Mx		NC-AJCC	pT4a N1b Mx LT-T_1_^0^ N-RLN^1^ PE-E^0^ (resection perichondrium one tracheal ring, sacrifice RLN, resection esophageal muscularis)
27	Yes	PTC-TCV	pT4a N1a Mx		NC-AJCC	pT4a N1a Mx LT-T_2_^0^ (resection perichondrium two tracheal rings)
28	Yes	Medullary	PT4a N1b Mx		NC-AJCC	pT4a N1b Mx PE-E^0^ (resection esophageal muscularis)
29	Yes	PTC-FV	pT4a N1a Mx		NC-AJCC	pT4a N1a Mx PE-E^0^ (resection esophageal muscularis)

PDTC, poorly differentiated thyroid carcinoma; PTC-FV, papillary thyroid carcinoma, follicular variant; PTC-TCV, papillary thyroid carcinoma, tall cell variant; PTC-TCF, papillary thyroid carcinoma, tall cell features; FC, follicular carcinoma; UFR, understaged final report; NCFR, no change in final report; NC-AJCC, no change in AJCC stage; R2, residual gross disease left in the patient; RLN, recurrent laryngeal nerve; PNI, perineural invasion.

In total, there were 14 final pathology reports for both surgeon A and B in which a checklist was not submitted. In all but one case, the disease process was understaged. A total of 15 cases (A and B combined) were accurately staged when accompanied by a surgeon-completed anatomic checklist (Fig. 1). There was a statistically significant improvement in the accuracy of staging as reported in the final pathology reports when an anatomic checklist was submitted as compared to when it was not (*P* < 0.01, Fisher exact test, two-tailed).

In the primary setting, the most commonly underreported structure was gross invasion of the pharyngoesophageal musculature (*n* = 8), followed by strap muscles (*n* = 7) and RLN sacrifice (*n* = 4). The cricothyroid muscle (*n* = 4) and tracheal perichondrium (*n* = 1) were the other structures missed in the primary cases.

In the six recurrent resections, gross nerve invasion requiring nerve sacrifice was not reported in four cases (RLN = 3 and vagus = 1). One recurrent case involved resection of the epineurium of the RLN (Table 2). All but one of the recurrences were located in the thyroid bed, leading to resection of all or a portion of the RLN in four cases, esophageal musculature in two, and laryngotracheal perichondrium in two. The pathology report for the one lateral compartment recurrence failed to report the resected carotid artery and vagus nerve. In all recurrent cases, a checklist was not incorporated, as it is not an obligatory reporting element to perform AJCC staging in recurrent cancer.

**Table 2:** Consecutive recurrent thyroid cancers with gross invasion, degree of structural involvement not reported.

Patient #	Surgeon	Diagnosis	Items not reported on	Recommended reporting and new classification
30	A	Recurrent PTC	RLN, SCM	Rec N-RLN^0^ M-SCM (recurrent cancer involving RLN epineurium, sternocleidomastoid muscle)
31	B	Recurrent PTC-TCV	RLN, esophagus	Rec PE-E^0^ N-RLN^1^ (recurrent cancer involving esophageal muscularis, RLN sacrifice)
32	B	Recurrent PTC	Esophagus, RLN or cricoid	Rec PE-E^0^ N-RLN^1^ LT-CR^0^ (recurrent cancer involving esophageal muscularis, RLN sacrifice, cricoid perichondrium)
33	B	Recurrent PTC	PNI	Rec N-RLN^1^ (recurrent cancer requiring RLN sacrifice)
34	B	Recurrent PTC	PNI or carotid artery	Rec N-Vag^1^ VAS-Acc^1^ (recurrent cancer requiring sacrifice vagus nerve and common carotid artery)
35	B	Recurrent PTC	Trachea	Rec-LT-T^0^_2_ (recurrent cancer involving tracheal perichondrium, two rings)

PTC, papillary thyroid carcinoma; PTC-TCV, papillary thyroid carcinoma, tall cell variant; RLN, recurrent laryngeal nerve; SCM, sternocleidomastoid; PNI, perineural invasion; Rec, recurrent cancer.

Both surgeon A and B reported that the time required to complete and send the checklist was as short as 30 s, and in no case did it require more than 90 s.

## Discussion

Most thyroid cancers are low risk, and accurate staging usually does not require details of the preoperative and intraoperative findings. Surgeons and pathologists are often lulled into routines that require a jolt when dealing with grossly invasive thyroid cancer. For surgeon B, patient 20 (Table 1) was the impetus for implementing the anatomic checklist. For surgeon A, the adoption of the checklist was delayed until patient 14, after which all invasive cases were accompanied by a checklist. We conclude from this investigation that the use of a checklist is the most time-efficient process for surgeon-pathologist informational handoff. We envision its adoption as an addition to conventional tumor node metastasis (TNM) classification (e.g. Table 1 and Fig. 9). Preoperative information that is often known by the surgeon and entered into a clinic note, such as the results of molecular testing and the status of vocal cord function, has significant clinical and prognostic implications and is included in the anatomic checklist.

The missing anatomic detail on the recurrent thyroid cancer cases again highlights the gap between what the surgeon identifies intraoperatively and what the pathologist can confirm histologically. We have since adopted the checklist for both primary and recurrent cancers that demonstrate involvement of adjacent structures. We believe that the inclusion of anatomic structures resected and/or the designation of macroscopic (R2) or microscopic (R1) disease left in the tumor bed is important information for all clinicians to understand, as well as radiologists who will interpret post-treatment surveillance scans. An example of a completed electronic output form is shown in Fig. 10. At the end of that online report, a table of AJCC staging and ATA ROR is included to help facilitate staging understanding and inclusion in the final pathology report.

Currently, the responsibility for identifying that a particular case requires intraoperative anatomic detail usually falls upon the pathologist. However, there is no standard regarding when pathologists must seek preoperative and intraoperative details. The limitations of non-surgeons, not present in the operating room, to accurately interpret the operative report for data required for accurate staging have been previously documented (5). It is related to both the failure to include that information in the operative note and the failure of the non-surgeons to interpret what is dictated. There is cognitive dissonance regarding the absence of an established, efficient mechanism for information transfer from surgeon to pathologist, in the face of requiring accurate AJCC staging and ATA ROR determination so vital for contemporary clinical management.

Optimal multidisciplinary therapeutic planning for patients with grossly invasive thyroid cancer requires consideration of many factors (2). Parsing out the degree of invasion into surrounding structures is important (4). Documenting the extent of resected disease, as well as disease left behind (R2), is also important and is usually not evident to a pathologist. Cancer can be dissected away from multiple structures without leaving any histological evidence and therefore may not be included in the final pathology report, reflecting the need for this anatomic checklist. Unlike the surgeon’s operative report, it is the final pathology report that follows patients throughout their thyroid cancer journeys. This important document should unambiguously reflect the true extent of the disease. The best opportunity to document the intraoperative extent of the disease is immediately after surgery when the details of surgery are fresh in the surgeon’s mind. The speed of checklist completion was viewed very favorably by both surgeons A and B. When compared to the time required by pathologists to obtain intraoperative data by either analyzing the surgeon’s operative report or the need to call the surgeon to obtain that information, the overall efficiency was very evident. The ability to automatically generate an electronic classification form that is devoid of patient identifiers and can be emailed to the pathologists efficiently completes the information transfer. In addition, the relative rarity of grossly invasive thyroid cancer creates the opportunity to establish a registry to promote multi-institutional collaboration and case accrual with outcome data, which would further our understanding of these advanced thyroid malignancies.

### Limitations

We acknowledge the inherent limitation of this initiative, which is the lack of outcome data related to this granular classification. We also acknowledge the limited number of patients presented in this series. From a patient quality-of-care point of view, it’s important to publicize this information gap to stimulate surgeons to improve communication with pathologists.

The fragmented and unindexed nature of operative reports renders retrospective data gathering impossible. Grossly invasive thyroid cancer demonstrating ETE or ENE is relatively rare. It will take many years to complete the necessary long-term prospective studies using this granular schema. The development, dissemination and adoption of a standardized checklist for recording these data are necessary steps that must precede a prospective outcome study. The anatomic sites selected for inclusion and the very need for this checklist emanated from our collective experience as senior thyroid surgeons and thyroid pathologists. While we propose the checklist as a quick and efficient means of communication, we understand that some surgeons may elect to provide detailed anatomic information in their operative notes. We encourage all surgeons who are reporting on the surgical procedures rendered in the setting of invasive thyroid cancer to adopt the convention of including a section on ‘key intraoperative findings’ at the beginning of their operative note, where critical information can be readily found by all non-surgeons. Accurate final staging requires each surgeon/pathologist team to create a systematic method of communication.

The format of subscripts and superscripts for five anatomic sites might initially appear daunting. The consistency of formatting across subsites makes these shorthand designations easy to manage, and the anatomic drawings facilitate data entry. Multi-institutional studies are needed to assess the ease of use of this approach as well as the willingness of surgeons/pathologists to adopt it. We also recognize that endorsement by national surgical and pathology societies will be a key element in helping to drive adoption.

### The case for adding anatomic detail

AJCC grouping of patients into T3b, T4a and T4b assumes that the prognosis for gross ETE differs only by the anatomic structures involved and is not influenced by the degree of involvement (3). This issue is unresolved and cannot be studied using present staging systems. The inherent limitations of the existing reporting system are evident if we analyze an example of RLN invasion (T4a). RLN invasion is usually reported when the surgeon resects the RLN. pT4a disease may be understaged if the specimen histology or the specimen part labeling does not convey the surgeon’s knowledge that the cancer involves the nerve. Surgeons reporting on structural involvement, such as the RLN, are not asked to distinguish between epineural involvement versus more extensive nerve encasement requiring sacrifice. Other examples include the lack of staging distinction between cancers dissected away from the larynx or trachea in a subperichondrial plane, or from the pharyngoesophagus involving the muscularis, versus those requiring more extensive structural resection. Overall, the ability to define, report and collect data regarding structural involvement in a more detailed fashion is the first step in determining if it is appropriate to lump patients together in the T3b, T4a and T4b classifications.

Thus, incorporating intraoperative findings to define T3b, T4a and T4b classification should carry over to the determination of other anatomic structures that assist in upstaging of disease. This is the mandate of the AJCC and the CAP and finds historical precedent in the work by Ito *et al.*, who proposed an ‘intraoperative staging system’ based on intraoperative findings (7).

As shown in some examples presented in Tables 1 and 2, we envision the addition of a suffix to the current TNM staging that reflects the extent of anatomic structural involvement in an easily understood designation. Some information in this system can potentially further stratify patients regarding the risk of death and recurrence. Granularity of information is imperative to discern the degree of involvement of a particular structure rather than the current binary determination. Unless a mechanism can report detailed information such as this, we will not be able to understand its significance. As Shin *et al.* demonstrated, the degree of penetration of the trachea impacts oncologic outcomes (8). The same may hold for other structures.

## Conclusion

The inherent complexity of invasive thyroid cancer mandates a multidisciplinary approach and requires a detailed understanding by all involved clinicians of the intraoperative findings and extent of surgery performed. Here, we demonstrate the benefit of an anatomic checklist to enhance surgeon-pathologist communication and improve final staging accuracy. The decision to include that checklist in the patient record will be made at an institutional level. This granular information regarding the precise location of residual disease and the location/extent of structural invasion will be invaluable for tracking surveillance scans and decision-making regarding adjuvant radiation therapy. This added value justifies its inclusion in the patient record and should accompany the permanent pathology report. We introduce a novel anatomic checklist with a convenient shorthand notation to provide uniformity. This classification system lends itself to data analysis, which could never be achieved by a pathologist gleaning information from the operative note.

The extent of anatomic detail will require further prospective multi-institutional studies to validate and/or modify the details included herein. Outcomes data are needed to determine the significance of including anatomic detail to the current TNM staging system. We hope that the collection of this data will promote and facilitate this type of research.

## Declaration of interest

M U, M B-W, M Z, A Shaha, Z B, R M T serve as consultants to TIRO. The authors declare that there are no conflicts of interest that could be perceived as prejudicing the impartiality of the research reported.

## Funding

This research idid not receive any specific grant from any funding agency in the public, commercial or not-for-profit sector.

## Author contribution statement

M U, M B-W contributed to conceptualization, writing original draft, data curation and formal analysis; R L C was responsible for data curation, formal analysis and reviewing and editing the manuscript; M Z, M S, J S, contributed to writing, reviewing and editing the manuscript; J F, A Silberzweig, J K J, contributed to reviewing and editing the manuscript; R G, A Shaha, Z B, R M T, contributed to conceptualization, reviewing and editing the manuscript.
